# Insight into the Stability of Cross-β Amyloid Fibril from VEALYL Short Peptide with Molecular Dynamics Simulation

**DOI:** 10.1371/journal.pone.0036382

**Published:** 2012-05-10

**Authors:** Wei Ye, Yue Chen, Wei Wang, Qingfen Yu, Yixue Li, Jian Zhang, Hai-Feng Chen

**Affiliations:** 1 State Key Laboratory of Microbial Metabolism, Department of Bioinformatics and Biostatistics, College of Life Sciences and Biotechnology, Shanghai Jiaotong University, Shanghai, China; 2 Shanghai Center for Bioinformation Technology, Shanghai, China; 3 Department of Pathophysiology, Key Laboratory of Cell Differentiation and Apoptosis of Chinese Ministry of Education, School of Medicine, Shanghai Jiaotong University, Shanghai, China; University of Akron, United States of America

## Abstract

Amyloid fibrils are found in many fatal neurodegenerative diseases such as Alzheimer's disease, Parkinson's disease, type II diabetes, and prion disease. The VEALYL short peptide from insulin has been confirmed to aggregate amyloid-like fibrils. However, the aggregation mechanism of amyloid fibril is poorly understood. Here, we utilized molecular dynamics simulation to analyse the stability of VEALYL hexamer. The statistical results indicate that hydrophobic residues play key roles in stabilizing VEALYL hexamer. Single point and two linkage mutants confirmed that Val1, Leu4, and Tyr5 of VEALYL are key residues. The consistency of the results for the VEALYL oligomer suggests that the intermediate states might be trimer (3-0) and pentamer(3-2). These results can help us to obtain an insight into the aggregation mechanism of amyloid fibril. These methods can be used to study the stability of amyloid fibril from other short peptides.

## Introduction

Amyloid fibrils are found in many fatal diseases, including Alzheimer's disease, Parkinson's disease, type II diabetes, and the transmissible spongiform encephalopathies [1]. These diseases are caused by the aggregation of disordered proteins. Therefore, the aggregation of misfolding protein plays a key role in these diseases [2]. X-ray experiment observes that the product of protein aggregation contains a cross-β spine and β-strands perpendicular to the fibril axis [3]. Moreover, the amyloid fibril is noncrystalline and insoluble. That is, it is difficult to crystalize atomic-level structures of cross-β spine with traditional experimental methods. Until 2007, Eisenberg's group released a set of crystal structures for amyloid-like fibril of short peptides from different protein precursors by X-ray microcrystallography [3]. These atomic-resolution structures make it possible to investigate the common characters of amyloid formation by molecular dynamics simulations, which can directly compare with experimental results [4].

However, the mechanism of fibril formation is poorly understood. To explain the transition of peptides from soluble to fibrous forms, several types of atomic-level models have been proposed, such as refolding, natively disordered and gain of interaction [3]. Oligomers or intermediate assemblies of protein are identified as the toxic agents that interact with cellular machinery [5]–[6]. To understand the kinetics of fibril formation and the molecular mechanism of transition from monomers to fibrils, the growth of amyloid fibrils and the self-assembly of multisubunit protein complexes are studied [7]. The self-assembly includes the stabilization of transient α-helices through the formation of NMR-invisible helical intermediates and conformational rearrangement from α-helix to β-sheet [8]. At the same time, there are also some computational studies to provide an insight into the characteristic of the short segments of the amyloid-like aggregation [9], [10], [11], [12], [13], [14], [15], [16], [17], [18], [19], [20], [21], [22], [23]. Toschi *et al* suggests that electric fields are favorable to the switch of Aβ-peptides from helical to beta-sheet conformational transition [24]. Masman *et al* explores the contributions of the different structural elements of trimeric and pentameric full-length Aβ(1–42) for the aggregation in solution [25]. Kent *et al* reports that a solvent-exposed hydrophobic patch is important for the aggregation of Aβ(10–35) [15]. Zheng *et al* studies Aβ40 elongation, association, and the aggregation pathway of β2-microglobulin amyloid with molecular dynamics simulations [26]. Sgourakis *et al* researches the flexibility of C terminus of Aβ42 which is responsible for the higher propensity to form amyloids [27]. DeMarco and Daggett study the aggregation process of prion fibril using atom molecular dynamics simulations [12]. Wu *et al* reports the time scale of aggregation for amyloidogenic hexapeptide NFGAIL [22]. Wang et al studies the disaggregation behaviour of GNNQQNY oligomer [28]. Furthermore, Gnanakaran *et al* investigates the aggregation of simple amyloid β-dimer with replica-exchange molecular dynamics [14]. Lin *et al* reports the structural stability and aggregation behavior of the VEALYL peptide [29]. These previous works can partly reveal the self-assembly mechanism of amyloid fibril. However, we still do not know if there is an intermediate state during the aggregation of different protein precursors. To shed light on this question, all atom molecular dynamics simulation was used to analyze the aggregation mechanism of VEALYL short peptide.

**Figure 1:**
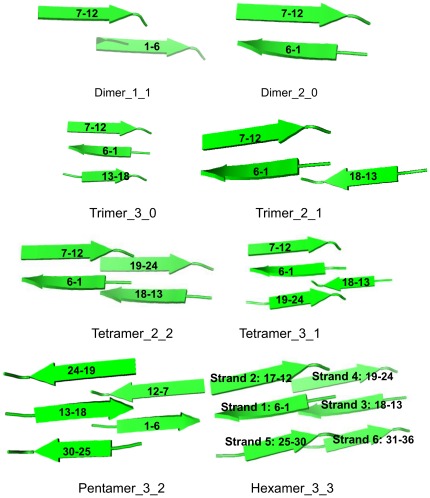
The schematic organization of dimer, trimer, tetramer, pentamer, and hexamer VEALYL model. The organization of strand is indicated.

In our previous work, we use molecular dynamics simulation to investigate the stability of of hexamer for eight class peptides. The MD results suggest that VEALYL and MVGGVV-1 are the most stable ones. Then we study the aggregation mechanism of MVGGVV-1 amyloid fibrils [30]. The results indicate that the study of short peptide aggregation could reveal some common fundamental mechanisms for the fibril formation in large protein systems. Therefore, in this study, we intend to research the stability of VEALYL peptide to understand its aggregation mechanism using room-temperature molecular dynamics simulation in explicit water. The VEALYL hexamer model was shown in Figure 1.

## Results and Discussion

### 1. The stability of VEALYL hexamer

The previous work suggests that a small number of trajectories for MD simulation (5–10) is sufficient to capture the average properties of the protein [31]. Therefore, 10 trajectories of 20.0 ns each were simulated at 298 K to analyze the stability of VEALYL hexamer. The Cα atom RMSD for representative trajectory was shown in supplement Figure S1. The RMSD was about 2.5 Å for VEALYL hexamer. This suggests that VEALYL hexamer became dynamics equilibration after 15.0 ns simulation.

**Figure 2:**
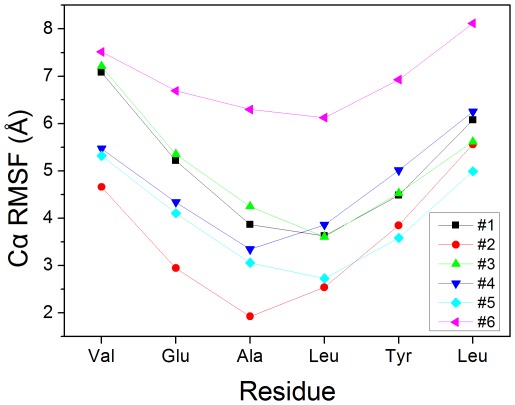
Cα variation of residues for VEALYL hexamer. Each short peptide is monitored, respectively. The fluctuations of six peptides are different.

To analyze the stability, the Cα fluctuations of VEALYL hexamer were illustrated in Figure 2. The figure indicates that all chains have common characteristics of small variation for the five central residues whereas large variation for the two end residues. This suggests that the center residues are more rigid than those in the termini region. This is in agreement with the results of Zheng et al. [32] However, the fluctuation of residues 1–2 was larger than that of residues 5–6 for strands 1 and 3, and the fluctuation for strands 2 and 6 was the reverse. The fluctuation of two termini residues for strands 4 and 5 had no significant difference. According to the asymmetric fluctuation, a little twist was found for beta-strand of VEALYL hexamer peptide during room temperature simulation. This is consistent with the results of other simulations [25], [33].

**Figure 3:**
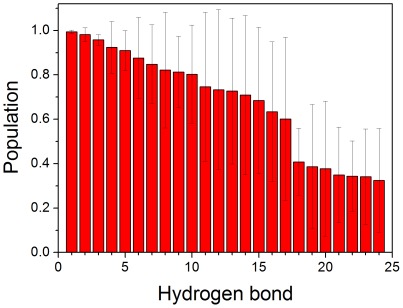
Hydrogen bond for VEALYL hexamer. Hydrogen bond was arranged by descending order according to the population. The populations of 17 hydrogen bonds are larger than 40%.

**Figure 4:**
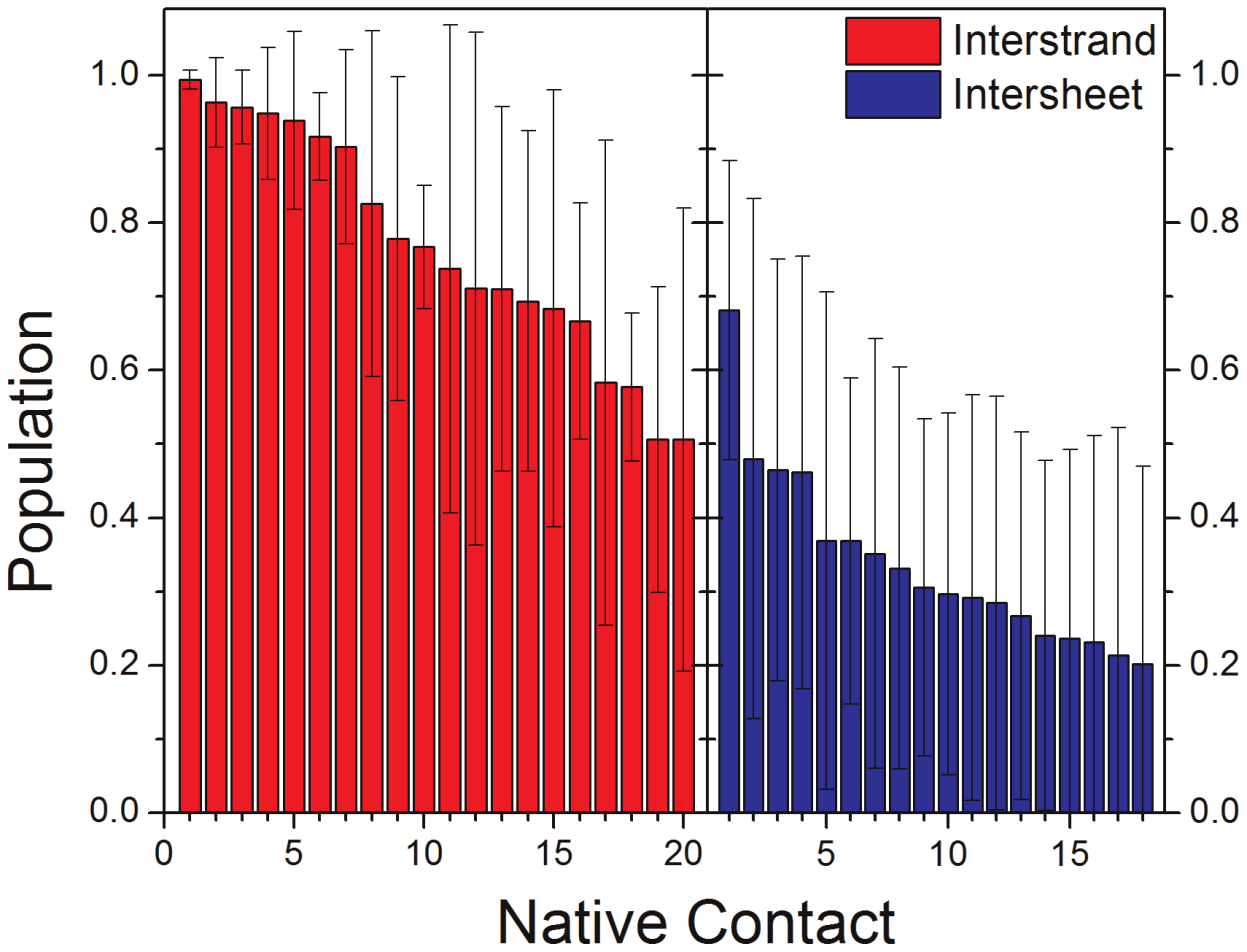
Native contact of VEALYL hexamer. Native contact was classed into interstrand and intersheet. Native contact was arranged by descending order according to the population. The native contacts of interstrand are stronger than those of intersheet.

To further study the driving force for the stability of steric zipper motif, the native contacts and hydrogen bonds for VEALYL hexamer were calculated. A hydrogen bond was assigned if the distance between donor and acceptor atoms was less than 3.5 Å. The populations of hydrogen bond for ten trajectories were shown in Figure 3. 17 stable hydrogen bonds were found, with populations higher than 40%. These hydrogen bonds played key roles in stabilizing the zipper motif. This is consistent with the previous observation that hydrogen bond was found in the 16 peptides of VEALYL [9]. Besides hydrogen bond, we also researched the native contact of VEALYL hexamer. There were two types of native contact. One was the contact of interstrand, and the other was that of intersheet. An intersheet chain contact was defined if the distance between the center mass of two side chains was less than 6.0 Å. The native contacts for intersheet and interstrand were shown in Figure 4. There were 20 and 4 stable native contacts for interstrand and intersheet with populations higher than 40%. The native contacts of interstrand focused on Val1, Leu4, and Tyr5. The native contacts of intersheet focused on Val1 and Leu4. In summary, these native contacts of interstrand and intersheet should be major driving forces for the aggregation of VEALYL peptide.

### 2. Mutant Research

**Figure 5:**
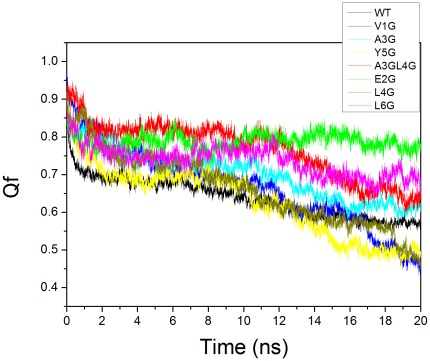
The fraction of native contact between β-strands for wild type and mutants during the 20 ns MD simulations. The color code of wild type and mutants (V1G, E2G, A3G, L4G, Y5G, L6G, and A3GL4G) was labeled in the caption.

The native contacts for VEALYL hexamer suggest that Val1, Leu4, and Tyr5 are key residues to stabilize the hexamer interface. To confirm the results of statistics analysis on these key residues, these residues were mutated to tiny Gly for each peptide, respectively. According to the distribution of these residues in sequence, mutant research could be classed into two categories: single-point and two linkage residue mutant. For wild type and mutant, some native contacts are between the side chains of two amino acids that are not neighboring in the amino acid sequence. The fraction of native contacts (Qf) was used as a reaction coordinate for measuring the deviation from the native state of structures produced during molecular dynamics simulations. The Qf for wild type and mutants was shown in Figure 5. The Qf of wild type was larger than 60% and kept constant during 20 ns simulation. This suggests that the wild type of VEALYL hexamer is very stable. This is consistent with the results of RMSF. For the single-point mutant, the Qf of V1G, L4G, and Y5G decreased during 20 ns simulation and is significant lower than that of the wild type, respectively. However, the Qf of E2G, A3G, and L6G is larger than that of WT. For two linkage mutant, the Qf of A3GL4G was slight higher than that of the wild type. This confirms that Val1, Leu4, and Tyr5 are key residues for the zipper stability.

**Figure 6:**
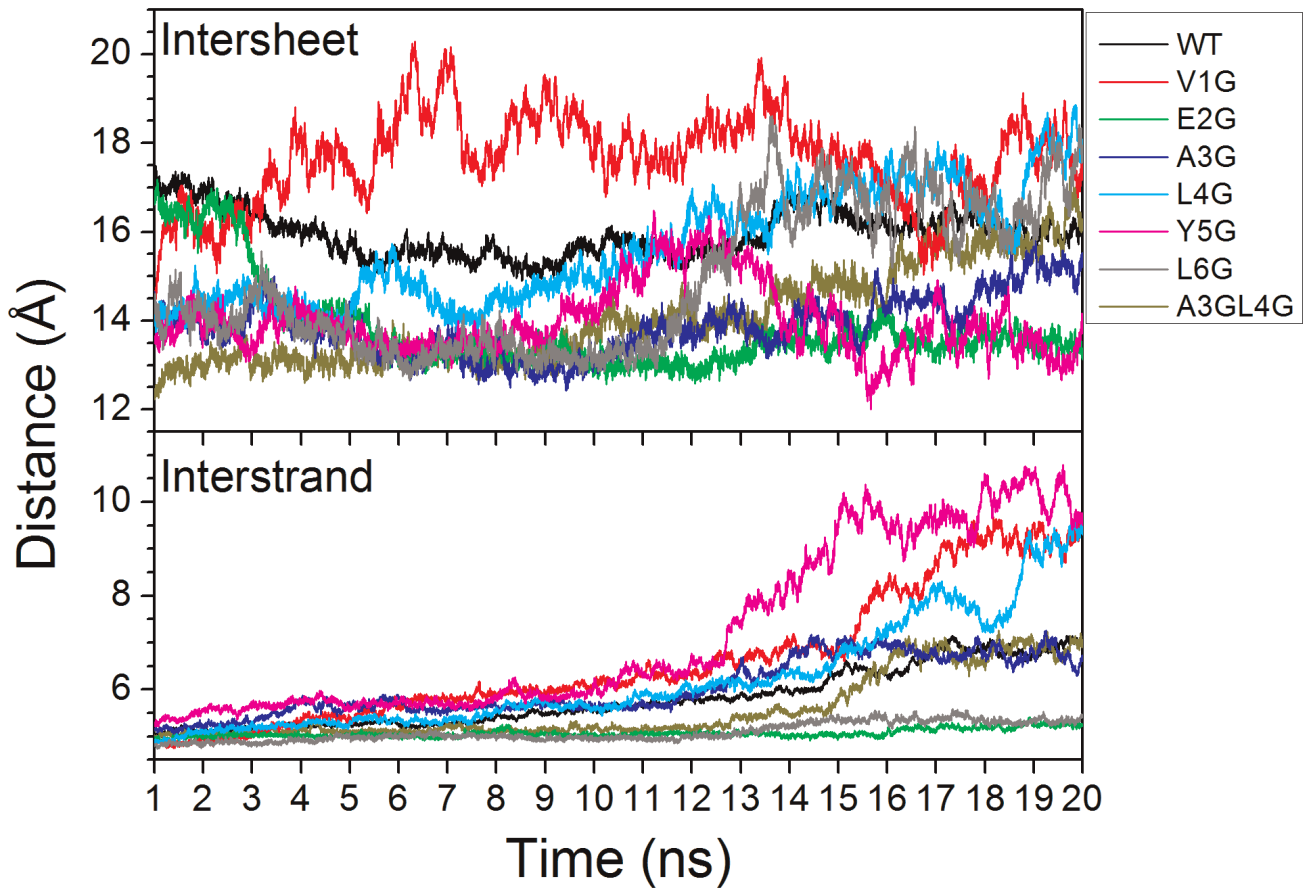
The average interstrand and intersheet distance between β-strands for wild type and mutants during 10 ns simulation. The interstrand distance was calculated by averaging pairwise residue Cα-Cα distances of strand1-strand2, strand2-strand3, strand4-strand5, and strand5-strand6 within the same β-sheet layer. The intersheet distance was calculated between the Cα center of sheet1 and sheet2.

The distances of interstrand and intersheet for wild type and mutants were shown in Figure 6. The distance of interstrand for WT, A3G, and A3GL4G was about 6 Å, respectively. The distance of interstrand for E2G and L6G was about 5 Å and their variations were very small. The distance of interstrand for V1G, L4G, and Y5G was about 9 Å and significant higher than that of WT. This suggests that the strands of V1G, L4G, and Y5G have the propensity of expansion. The distance of intersheet for WT was between 16 Å and 17 Å. Surprisingly, the distance of intersheet for E2G, A3G, and Y5G decreased relative to WT. The β-sheets have the propensity of compression. This suggests that E2G and A3G might play some roles in the stability of the fibril [29]. On the contrary, the distance of intersheet for V1G and L4G significantly increased. The hexamers of V1G, L4G, and Y5G are almost disaggregation. The mutant of V1G, L4G, and Y5G will lead to the disaggregation of hexamer.

**Figure 7:**
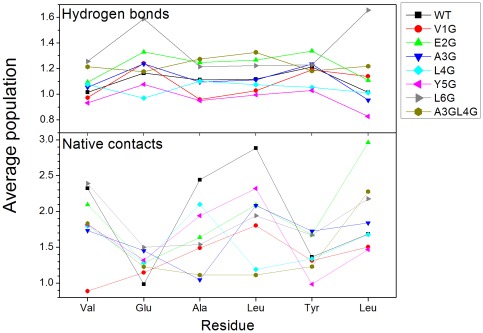
The average number of native contact and hydrogen bond for each residue of wild type and mutants.

To reveal the structural adjustment for mutants, the interactions between these short peptides were shown in Figure 7. The average native contact of each residue for V1G, L4G, and Y5G is 1.36, 1.57, 1.64, and lower than that of WT (1.95), respectively. This suggests that the mutant of Val1, Leu4, and Tyr5 decreases the native contact between residues. For hydrogen bond, the average number for V1G, L4G, and Y5G was 1.09, 1.05, 0.97, and lower than that of WT (1.11), respectively. The average hydrogen bond for A3G was 1.11 and similar to that of wild type. The average hydrogen bond for E2G, L6G, A3GL4G was 1.23, 1.36, 1.23, and larger than that of WT, respectively. This suggests that the mutant of Val1, Leu4, and Y5G decreases the hydrogen bonds of residue. In summary, Val1, Leu4, and Tyr5 were key residues for VEALYL aggregation combined hydrogen bond and native contact analysis.

To study the stability of mutation, Cα variations of wild type and mutants were illustrated in supplement Figure S2. The RMSF of V1G was the largest. The RMSF of L4G and Y5G was also higher than that of WT, respectively. The RMSF of A3G and L6G was similar to that of wild type. The variation order of RMSF for V1G, L4G, and Y5G was consistent with the result of distances for intersheets and interstrands.

### 3. Comparison for the stability of oligomer

**Figure 8:**
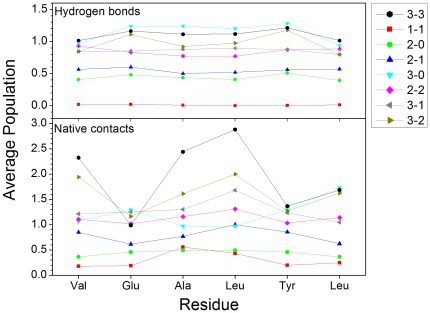
Average number of native contact and hydrogen bond for each residue of oligomer. Native contact between pairwise residues was calculated, then added according to the residue number of normalized peptides, and divided by the number of peptide. Hydrogen bond was calculated, then added according to the residue number of normalized peptides, and divided by the number of peptide.

In order to reveal the aggregation kinetics, the stabilities of dimer, trimer, tetramer, and pentamer were studied. The simulation condition was also gathered in Table 1. As shown in supplement Figure S3, the Cα RMSDs quickly increased to 25 Å for dimer (1-1) and ∼10 Å for dimer (2-0) after 15 ns simulation. This suggests that the dimer is not stable and discards their original organization of structure during 20 ns simulation. This is consistent with the result of Zheng et al that the dimer of GNNQQNY is not thermodynamically stable state [32]. Their average structures absolutely depart from the initial coordination of dimer. The β-sheet structure of dimer (2-0) also discarded. Then, how about the stability of the trimer with addition a strand based on the dimer? The trimer (2-1) was neither stable and its RMSD was about 20 Å after 15 ns simulation. However, the RMSD of trimer (3-0) was about 2.5 Å. The average free energy of trimer (3-0) and (2-1) was calculated with MMPBSA [34]. The average energy was −677.70±13.73 kcal/mol for trimer (3-0) and −657.31±14.64 kcal/mol for trimer (2-1). The relative energy difference between trimer (3-0) and trimer (2-1) is about −20.39 kcal/mol. This indicates that the trimer (3-0) was a relative stable state to trimer (2-1). *Gsponer et al.* have reported that three-stranded parallel in-register aggregates as nucleus from three peptides simulation in an implicit solvent [35]. For the model system of the tetramer with addition another strand, the stability of tetramer (2-2) was similar to that of trimer (2-1). Their RMSDs were about 20 Å. This suggests that tetramer(2-3) and trimer (2-1) are unstable. The RMSD of pentamer (3-2) was similar to that of wild type, indicating that the pentamer (3-2) is a stable state. Experimental observation also confirms this point [5]. The residue fluctuation of these oligomers was shown in supplement Figure S4. The fluctuations of trimer (3-0) and pentamer (3-2) were the smallest among these oligomers. The average native contacts and hydrogen bonds for these oligomers were shown in Figure 8. The average number of hydrogen bond for trimer (3-0), pentamer (3-2), and hexamer(3-3) was the largest among these oligomers. The result was consistent with the result of RMSF. According to their stabilities, the intermediate states should be trimer (3-0) and pentamer (3-2). This is in agreement with the other research that pentamer(3-2) and trimer (3-0) may serve as a minimal nucleus seed for the formation of the VEALYL fibrils [29]. Collins *et al* report that fibers grow by monomer addition [36]. For tetramer (3-1), it also has large average values of hydrogen bond and native contacts. This suggests that the agregation of pentamer (3-2) might be through tetramer (3-1) and trimer(3-0).

**Table 1:** Simulation condition of wild type and mutant for VEALYL peptides.

Type	Monomer sequence	strand/sheet organization	counter ions	water	trajectory	Time(ns)
Oligomer	VEALYL	Dimer_1_1	/	1270	5	100
	VEALYL	Dimer_2_0	/	1270	5	100
	VEALYL	Trimer_2_1	/	1905	5	100
	VEALYL	Trimer_3_0	/	1905	5	100
	VEALYL	Tetramer_2_2	/	2543	5	100
	VEALYL	Tetramer_3_1	/	2543	5	100
	VEALYL	Pentamer_3_2	/	3178	5	100
	VEALYL	Hexamer_3_3	6 Na+	3813	10	200
Mutant	GEALYL(V1G)	Hexamer_3_3	/	3815	5	100
	VGALYL(E2G)	Hexamer_3_3	6 Na+	3813	5	100
	VEGLYL(A3G)	Hexamer_3_3	6 Na+	3811	5	100
	VEAGYL(L4G)	Hexamer_3_3	6 Na+	3815	5	100
	VEALGL(Y5G)	Hexamer_3_3	6 Na+	3811	5	100
	VEALYG(L6G)	Hexamer_3_3	6 Na+	3810	5	100
	VEGGYL(A3GL4G)	Hexamer_3_3	6 Na+	3811	5	100

## Methods

### 1. The definition of oligomer

The initial atomic coordinates of VEALYL hexamer were constructed from crystal structure 2OMQ using the symmetry operation *P*21 [3]. Single point mutation for V1G, E2G, A3G, L4G, Y5G, L6G and two linkage mutant for A3GL4G of hexamer were built with SCWRL3 [37]. For these hexamers, the native contacts are focused on the interfaces of amyloid fibril and classed into two categories. One is from inter-strands, such as between strands 1 and 2, strands 2 and 3, strands 4 and 5, strands 5 and 6. The other is from inter-sheets, such as between strands 1 and 4, strands 1 and 5, strands 2 and 5, strands 2 and 6, strands 3 and 6 (shown in Figure 1). According to the arrangement of short peptides, there is one possibility for hexamer and pentamer. For tetramer, there are two possibilities, one three strands in one sheet and the fourth on the other sheet, or each two in the same sheet (3-1 *versus* 2-2). For trimer, there are also two possibilities, all three strands belong to one single sheet, or two strands in one sheet and the third on the other sheet (3-0 *versus* 2-1). For dimer, two conformers are defined. Two strands are on one sheet or belonged to different sheets (2-0 *versus* 1-1). The atomic coordinates of these oligomers were constructed and extracted from VEALYL hexamer. The VEALYL dimer, trimer, tetramer, and pentamer models were also shown in Figure 1.

### 2. Molecular dynamics simulation

Hydrogen atoms were added using the LEAP module of AMBER8 [38]. Particle Mesh Ewald (PME) [39] was employed to treat long-range electrostatic interactions with the default set in AMBER8. The wild type of hexamer model was solvated in a truncated octahedron box of 3813 TIP3P water molecules so that the final concentration of the system after equilibration is 98 mM, which is at the upper end of the experimental concentration range for crystallization [3]. Other systems were solvated according to the condition of Table1. A revised parm99 force field was used for intramolecular interactions [40]. 1000-step steepest descent minimization was performed to relieve any structural clash in the solvated system. The SHAKE algorithm [41] was employed to constrain bonds involving hydrogen atoms so that a 2 fs time step was used. The minimized system was heated up and equilibrated in the NVT ensemble at 298K with PMEMD of AMBER8. Langevin dynamics was used in the heat and equilibration runs with a friction constant of 1 ps^−1^. Multiple independent trajectories of 20.0 ns each in the NPT ensemble at 298K were then simulated with PMEMD of AMBER8. The protocol is also shown in the literature [30], [42], [43], [44], [45], [46], [47], [48], [49]. The detail simulation condition was also listed in Table 1. A total of 1.6 µs trajectories were collected, taking about 67,920 CPU hours on the in-house Xeon (1.86 GHz) cluster.

### 3. The definition of stability

The stability of the aggregates was quantified by Cα RMSD. The parameter RMSD provides a measure of the deviation of the aggregate from the initial structure. The structures with Cα RMSD>5 Å were visibly disordered and thus were defined as unstable.

### 4. Data anlysis

The root mean square fluctuation (RMSF) is a measure of the deviation between the position of certain residue and initial reference position. Qf is the fraction of native contact during the simulation. Hydrogen bond is defined that the distance between two polar heavy atoms from different strands are less than 3.5 Å. The native contact is a contact between the side chains of two nonadjacent residues in hexamer's native state when their distance is closer than 6.5 Å. Native contact between pairwise residues was calculated, then added according to the residue number of normalized peptides, and divided by six peptides. Hydrogen bond was calculated, then added according to the residue number of normalized peptides, and divided by six peptides.

The free energies of molecules in solution were calculated with MM-PBSA [34].

## Supporting Information

Figure S1 **Cα RMSD of VEALYL hexamer for representative trajectory during 20 ns simulation.**
(TIF)

Figure S2 **The average Cα RMSF for wild type and mutants. The RMSF of V1G was the highest among these mutants.**
(TIF)

Figure S3 **Cα RMSD of eight VEALYL oligomers** ***versus*** **simulation time.**
(TIF)

Figure S4 **The average Cα variation of residues for each oligomer.**
(TIF)
