# Supplementary figures and images for: Insight into the Stability of Cross-β Amyloid Fibril from VEALYL Short Peptide with Molecular Dynamics Simulation
Source: PLoS One. 2012 May 10;7(5):e36382. doi: 10.1371/journal.pone.0036382 (PMC3349666; doi:10.1371/journal.pone.0036382)

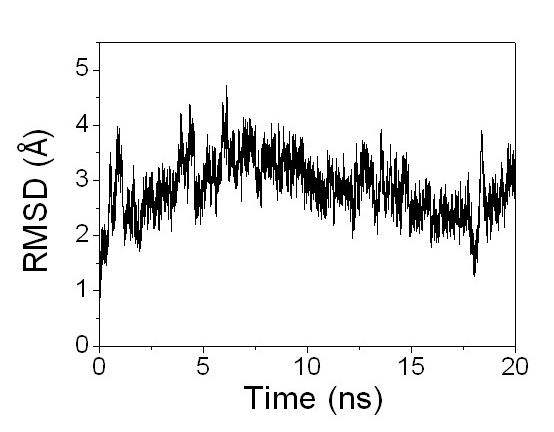

Supplement: Figure S1 — Cα RMSD of VEALYL hexamer for representative trajectory during 20 ns simulation. (TIF) [file pone.0036382.s001.tif]

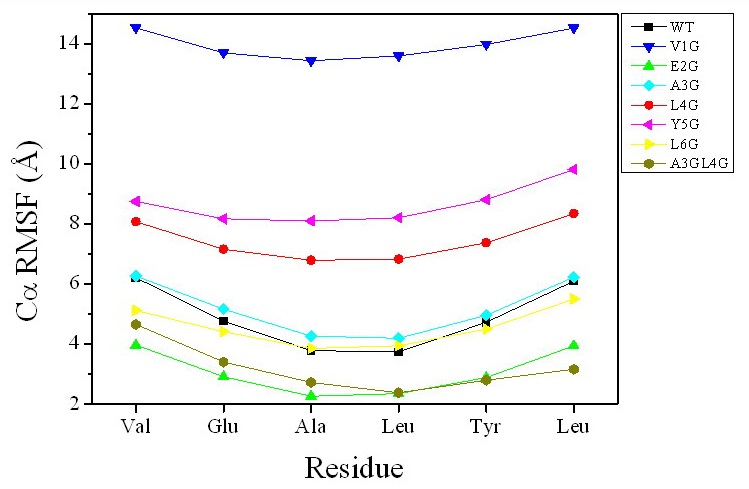

Supplement: Figure S2 — The average Cα RMSF for wild type and mutants. The RMSF of V1G was the highest among these mutants. (TIF) [file pone.0036382.s002.tif]

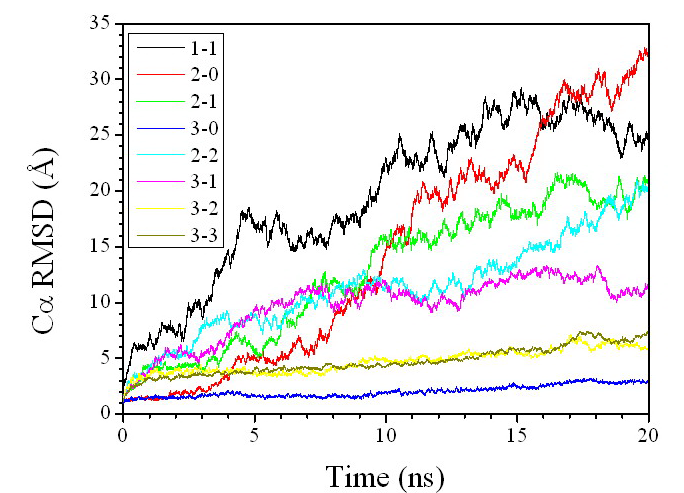

Supplement: Figure S3 — Cα RMSD of eight VEALYL oligomers versus simulation time. (TIF) [file pone.0036382.s003.tif]

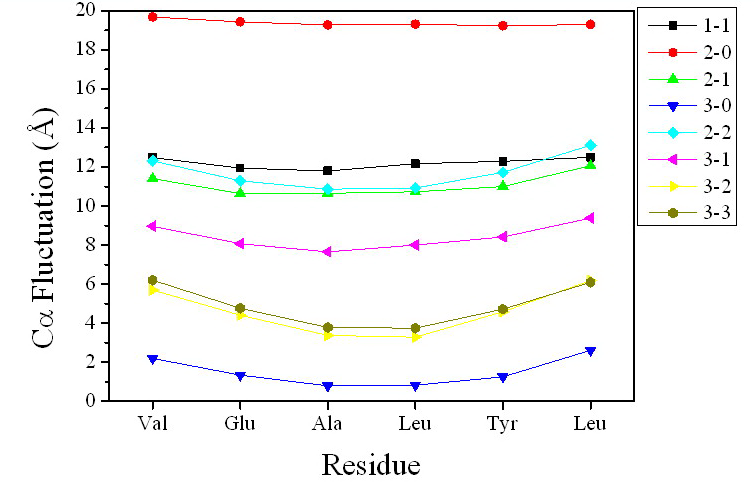

Supplement: Figure S4 — The average Cα variation of residues for each oligomer. (TIF) [file pone.0036382.s004.tif]
